# Extended Near-Infrared Optoacoustic Spectrometry for Sensing Physiological Concentrations of Glucose

**DOI:** 10.3389/fendo.2018.00112

**Published:** 2018-03-19

**Authors:** Ara Ghazaryan, Saak V. Ovsepian, Vasilis Ntziachristos

**Affiliations:** ^1^Institute for Biological and Medical Imaging, Helmholtz Zentrum München, German Research Centre for Environmental Health, Neuherberg, Germany; ^2^Munich School of Bioengineering, Technische Universität München, Munich, Germany

**Keywords:** glucose sensing, optoacoustic, near-infrared, diabetes, carbohydrate metabolism

## Abstract

Glucose sensing is pursued extensively in biomedical research and clinical practice for assessment of the carbohydrate and fat metabolism as well as in the context of an array of disorders, including diabetes, morbid obesity, and cancer. Currently used methods for real-time glucose measurements are invasive and require access to body fluids, with novel tools and methods for non-invasive sensing of the glucose levels highly desired. In this study, we introduce a near-infrared (NIR) optoacoustic spectrometer for sensing physiological concentrations of glucose within aqueous media and describe the glucose spectra within 850–1,900 nm and various concentration ranges. We apply the ratiometric and dictionary learning methods with a training set of data and validate their utility for glucose concentration measurements with optoacoustics in the probe dataset. We demonstrate the superior signal-to-noise ratio (factor of ~3.9) achieved with dictionary learning over the ratiometric approach across the wide glucose concentration range. Our data show a linear relationship between the optoacoustic signal intensity and physiological glucose concentration, in line with the results of optical spectroscopy. Thus, the feasibility of detecting physiological glucose concentrations using NIR optoacoustic spectroscopy is demonstrated, enabling the sensing glucose with ±10 mg/dl precision.

## Introduction

Optical spectroscopy (OS) is a widely employed method for sensing and assessment of the composition and physical properties of a sample of interest. A variety of optical spectroscopic methods have been developed based on different excitation and detection principles, operational range, as well as the scope of application. Among these, ultraviolet (UV), visible (Vis), near-infrared (NIR), middle infrared (MIR), Fourier transform infrared (FT-IR), fluorescence, and Raman spectroscopy is most commonly employed (1, 2).

Optoacoustic spectroscopy (OAS) represents a variant of OS which uses an acoustic (ultrasound) detector as opposed to an optical sensor (3). In OAS, ultrasound signals are generated within the specimen upon molecular absorption of transient light energy (3, 4). This method is typically employed to resolve light absorption in scattering (non-transparent) environment or intra-vital measurements, i.e., samples that are not well suited for conventional OS methods (5–7). In quantitative biology, OAS has been considered for sensing protease activity, tissue metabolites, nanoparticles, and biopolymers (8). Its use in studying nano-sized magnetic particles for drug delivery systems (9) or investigating the molecular structure and dynamics of DNA-ligand complexes and aromatic amino acids in peptides has been also demonstrated (9, 10). Furthermore, OAS has been applied for measurement of blood glucose levels in the MIR (11–14), taking advantage of the fact that glucose absorption offers distinct signatures in the so-called fingerprint region between approximately 800 and 1,200 cm^−1^ (12, 13). Nevertheless, MIR OAS readouts are limited by strong water absorption that hampers the analysis of large aqueous volumes and especially biological samples, confining the optical penetration to the surface (<100 μm) of the specimen (15).

In this study, we developed an optoacoustic spectrometer and for the first time investigate its ability to sense and retrieve spectra of glucose within physiologically relevant concentration ranges in the extended NIR range spanning 800–1,900 nm. We demonstrate that within this spectral range, OAS offers highly sensitive and accurate reads of physilogical glucose concentrations, which are in general agreement with those of conventional OS. Finally, we investigate the utility of different spectral processing methods and examine the wavelength range that may be best suited for glucose differentiation within the extended NIR.

## Materials and Methods

### Experimental Setup

**Figure 1:**
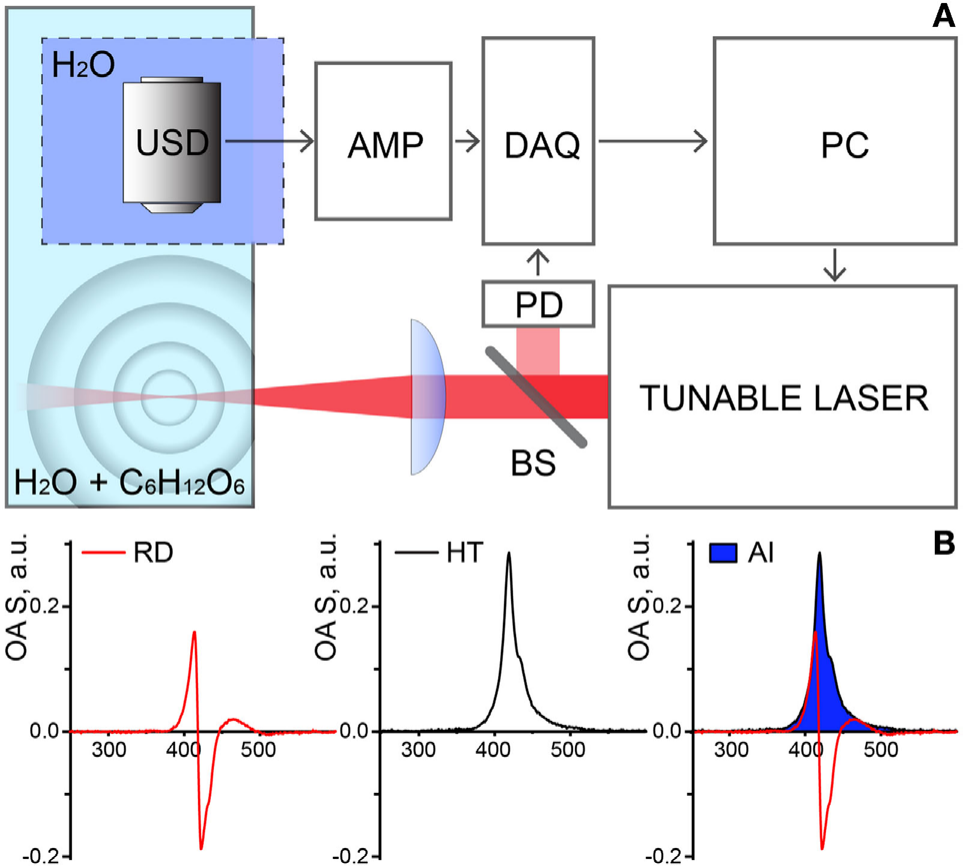
**(A)** Schematic representation of the experimental setup for optoacoustic spectroscopy (OAS). Abbreviations: USD, ultrasound transducer; DAQ, data acquisition card; PD, photodiode; BS, beam splitter; AMP, amplifier; PC, personal computer. **(B)** Stages of the data processing and analysis. OAS raw data (RD, left) is transformed with Hilbert transformation (HT, middle) and the area of interest (AI, right—in blue) under curves were taken as the measure of the OAS signal (*I*_OA_).

Optoacoustic measurements were performed within the extended NIR range of 850–1,900 nm using a custom built OAS (Figure 1A). Illumination was provided by a tunable nanosecond SpitLight Single OPO laser (Innolas, Krailling, Germany) controlled by a personal computer (PC). The output power was wavelength-dependent and ranged between 0.5 and 20 mJ. The wavelength scanning step was set to 25 nm. Upon the exit from the laser, the beam was first split by 95/5 beam splitter (BS), to reflect 5% to a photodiode (PD), used for triggering of detection and power-per-pulse registration; the rest of the beam was focused in the test chamber containing the 400 ml sample solution (distilled water with incrementing concentrations of glucose). The illumination beam was arranged perpendicular to the free surface of the solution with a focal point at ~3 mm depth. A cylindrically focused ultrasound detector (USD) with central frequency 15 MHz (V319, Olympus Panametrics-NDT, Tokyo, Japan) was immersed into a reservoir separated from the test chamber by a transparent plastic foil, to avoid deposition of the sampling material on the detector. The USD was adjusted to accommodate the beam in its focus. Acquired signals first were amplified with a low noise amplifier, AMP (AU-1291, Miteq Inc., USA), then digitized using a fast data acquisition card (DAQ) operating at 100 MS/s (EON-121-G20, Gage-applied, Lockport, IL, USA). The temperature of the solutions in the test chamber was maintained constant at 21°C.

### Glucose Measurements

A stock solution containing 1,200 mg/dl of D(+) Glucose (C_6_H_12_O_6_, Merck, Darmstadt, Germany) in distilled H_2_O was prepared immediately before the experiment and was added to the test chamber in 6 titrations of ~28 mg/dl, covering a concentration range of 0–170 mg/dl. The test chamber from the onset contained 400 ml DH_2_O. With each step of titration, aliquots of 10 ml solution were first dispensed from the test reservoir, which was followed by addition of 10 ml of concentrated glucose stock solution. Before each measurement, the content of test chamber was stirred for 5 min.

To investigate the ability to characterize glucose concentration from the spectra obtained, we first reduced the dimensionality of the NIR-OAS datasets by applying principal component analysis (PCA). PCA resolves a number of spectral principal components constituting the collected optoacoustic spectra and sorts the components according to their contribution strength on spectral variability. This approach enables elimination of measurement errors and drift-induced effects, which contribute to noise and systematic errors in the measurements (16).

Then, we compared two alternative analysis methods to measure glucose concentration from the PCA-processed spectra. The first method was based on sparse dictionary learning (SDL), which finds values of Δ*I*_OA_, corresponding to unit concentration of analyte (Δ*i*_U_) from a set of training solutions (TS) with known concentrations. Δ*i*_UC_ serves as the dictionary, based on which one can estimate the concentration of probe solutions. To do so we first found the least-squares solution to the system of equations

(1) $$\Delta i_{\text{UC}}*C_{gl}^{\text{TS}}=\Delta I_{\text{OA}}^{\text{TS}},$$

where $C_{gl}^{\text{TS}}$ is the vector containing set of known glucose concentrations of TS, $\Delta I_{\text{OA}}^{\text{TS}}$ is the matrix of Δ*I*_OA_ values, corresponding to each concentration of a training set. Subsequently, to estimate *C_gl_* for each Δ*I*_OA_ of probe set, a least square solution of Δ*i*_UC_*C_gl_ = Δ*I*_OA_ was calculated.

In addition to the SDL analysis, we investigated the ratiometric processing of the spectral data collected, as a method to identify glucose concentration in solution. Dual-wavelength ratiometry may afford estimation of a target substance independent of confounding factors introduced by the medium and/or experimental setup. Ratiometric analysis of glucose concentration *C_gl_* was computed assuming a ratio $R=I_{\text{OA}}^{\lambda_{1}}/I_{\text{OA}}^{\lambda_{2}}$ at two different wavelengths λ_1_ and λ_2_, respectively. Higher sensitivity is expected at selected wavelengths, where the glucose spectrum may have a prominent difference from the contributions of the background medium. The relationship between and the experimentally measured *R* is described by the calibration equation given by Grynkiewicz et al. (17)

(2) $$C_{gl}=K\frac{R-R_{0}}{R_{gl}-R},$$

where *R* is the experimentally measured ratio of *I*_OA_ at two wavelengths, *R*_0_ is the ratio at *C_gl_* = 0, *R_gl_* corresponds to the ratio *I*_OA_ of the known glucose spectrum at chosen wavelengths and *K* is a gain factor that can be defined with pre-calibration by fitting Eq. 2 to the data obtained from a set of solutions with known glucose concentrations. A set of five optoacoustic spectra was randomly selected for each concentration to represent the TS and used for calibration of the parameter *K*.

### Data Collection and Processing

Data acquisition and processing were performed in MATLAB (Mathworks, Natick, MA, USA). All signals were filtered using a fourth order exponential filter prior to analysis. Obtained values were normalized per laser energy registered by the PD to minimize errors related to laser pulse-per-pulse intensity fluctuations. The strength of the optoacoustic signal (*I*_OA_) was estimated by computing the Hilbert transform of the recorded acoustic signal of each measurement and integrating the area under the curve (Figure 1B) as previously reported (18). To obtain OA spectra of glucose, *I*_OA_ was measured for each glucose titration within the range of 850–1,900 nm with an incrementing step of 25 nm. For each concentration, 5 spectra of 200 measurements at each wavelength were recorded and averaged. The signal to noise ratio (SNR) was defined as a ratio of the mean value to standard deviation (SD) of the five values. The estimates of *C_gl_* obtained using the ratiometric and SDL methods were contrasted to the known glucose concentration values. For each concentration, we tabulated 5 *C_gl_* estimates corresponding to each of the 5 spectra collected. Pearson correlation coefficient, *r*, between estimated and expected values was calculated for both SDL and ratiometric methods.

## Results

**Figure 2:**
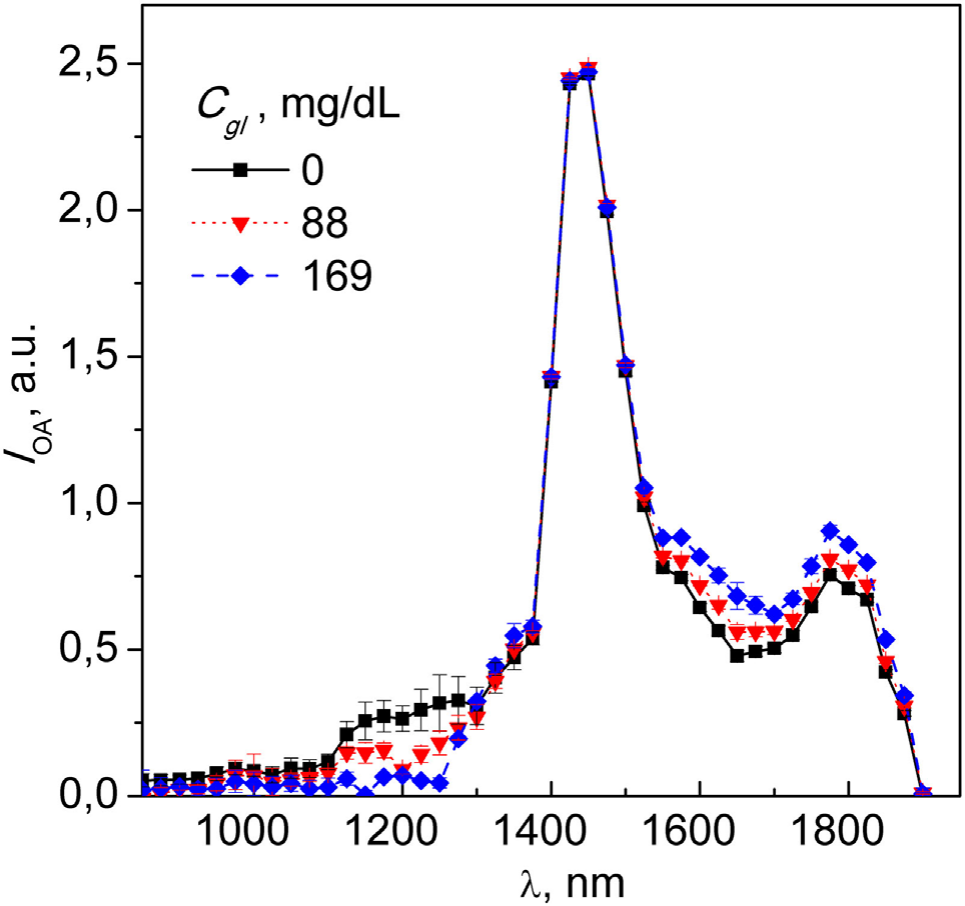
Spectra of distilled water with subsequent glucose titrations at 88 and 169 mg/dl; each spectral point represents an average of five independent readouts, with error bars indicating the SD of the optoacoustic signal. Note the dose-dependence diversion of the spectra of glucose-containing analyte from that of DH_2_O.

Figure 2 shows NIR spectra of the optoacoustic signal intensity (*I*_OA_) of distilled water and two concentrations of glucose in aqueous solution (88 and 169 mg/dl). This concentrations cover the physiological (healthy: 70–90 mg/dl fasting and 120–140 mg/dl after meal) and pathological (diabetic: 80–130 mg/dl fasting and 160–180 mg/dl after meal) ranges and are of relevance of potential medical application of our method. Reassuringly, the optoacoustic spectrum of water corresponds to data reported previously, with a peak absorption at ~1,450 nm (19). The spectral regions undergoing most pronounced changes upon addition of glucose are confined between 1,100–1,300 and 1,500–1,700 nm. Intensity variations seen in the five spectra collected for each glucose concentration are shown by the SD also plotted on Figure 2. The intensity variation observed could be partly attributable to the pulse-per-pulse energy fluctuations of the light source. Accordingly, the average SNR value calculated for each spectrum was estimated to be ~31.6.

**Figure 3:**
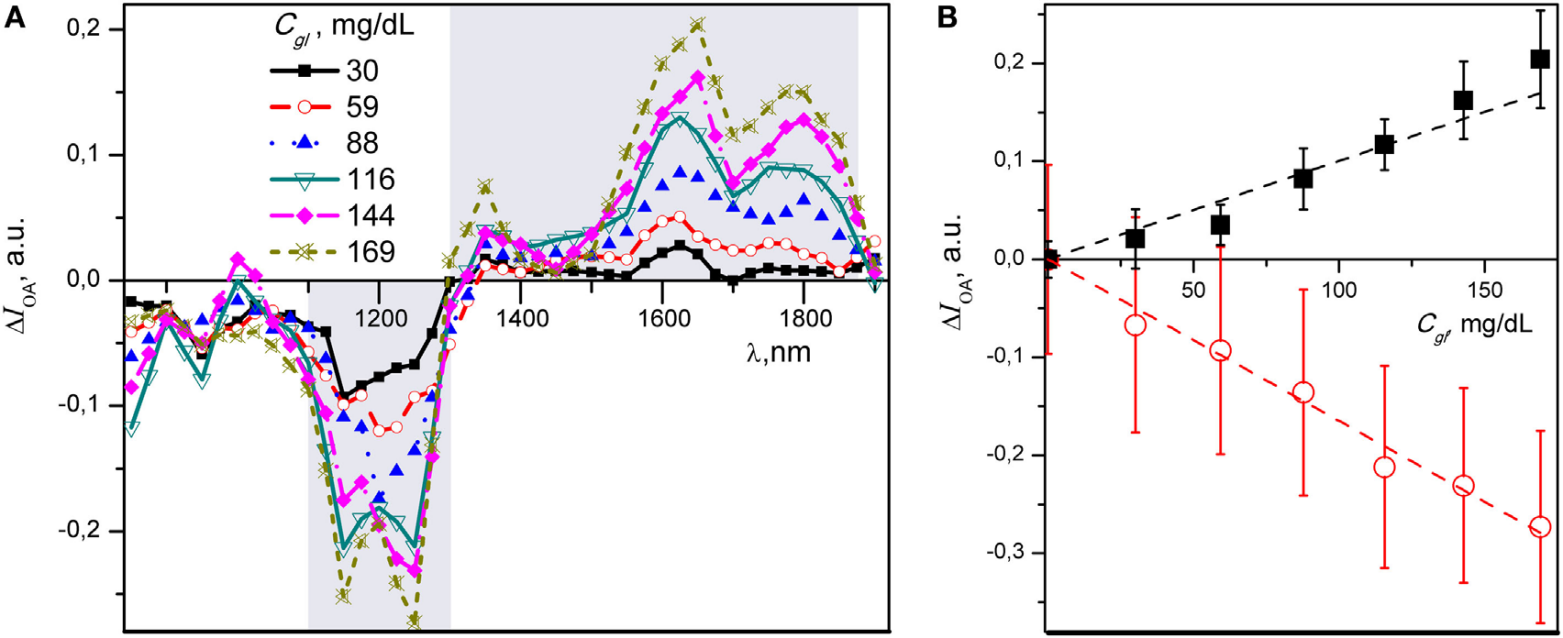
**(A)** Differential near-infrared (NIR) optoacoustic spectra of various glucose concentrations in aqueous media. Note negative and positive changes of the NIR optoacoustic spectral absorption. Each data point is a mean of five independent measurements. **(B)** Summary graph of the intensity—concentration relationship for Δ*I*_OA_ of glucose estimated at 1,250 nm (red) and 1,650 nm (black) wavelength with linear fits. Mean of five individual measurements are presented with SD.

To better visualize spectral changes due to glucose titration, we calculated the difference of optoacoustic signal intensity (Δ*I*_OA_) between each glucose containing spectrum and the baseline spectrum obtained from distilled water. Figure 3A presents mean values of Δ*I*_OA_ for six different glucose concentrations plotted over the 850–1,900 nm spectral range. For illustration purpose, the two spectral regions with most pronounced absorption differences are highlighted in gray.

The minimum of the negative region of NIR-OAS falls within 1,150–1,250 nm range, whereas two maxima of the glucose spectrum occur at 1,650 and 1,800 nm, respectively. The maximum absorption change observed throughout the spectra corresponding to 1,250 and 1,650 nm. These two wavelengths were subsequently selected for ratiometric analysis. The Δ*I*_OA_ at 1,250 and 1,650 nm demonstrates a closer linear relationship with *C_gl_* in both negative and positive directions, albeit with steeper slope in negative direction (Figure 3B).

**Figure 4:**
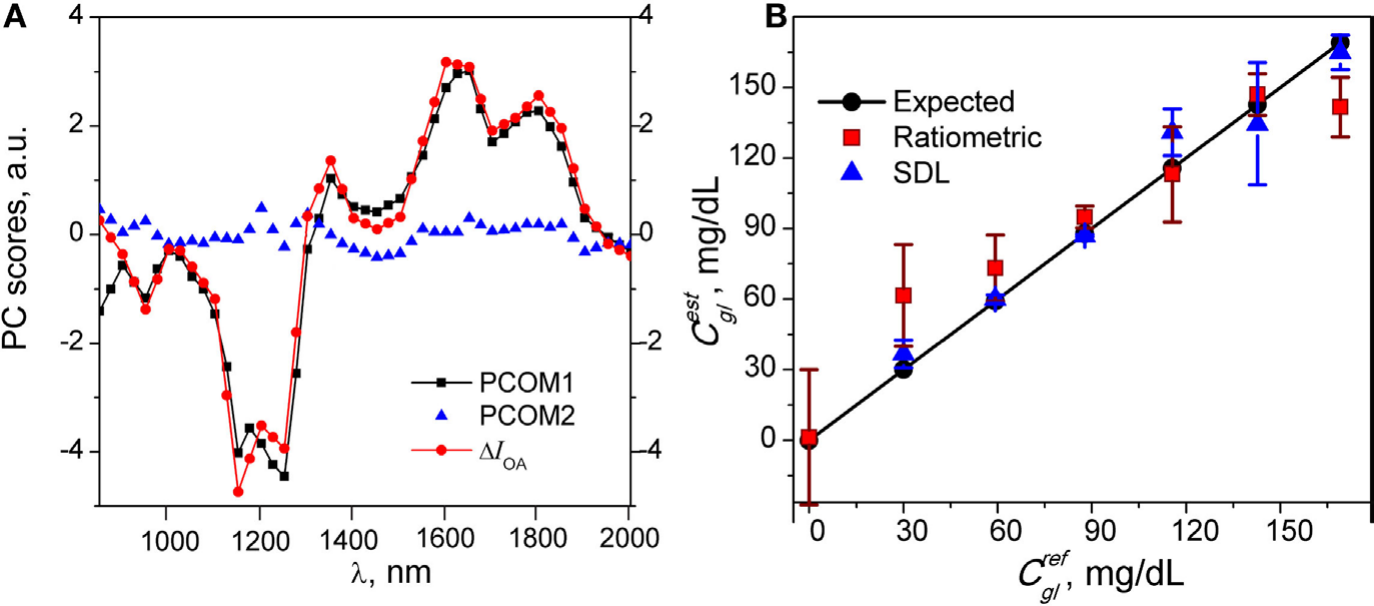
**(A)** Scores of PCA component (PCOM1) (black squares) and PCOM2 (blue triangles) of principal component analysis of the optoacoustic spectroscopy (OAS) of glucose. For illustration purpose, PCOM readouts are overlaid with near-infrared-optoacoustic spectroscopy (NIR-OAS) Δ*I*_OA_ (red circles) of 169 mg/dl glucose solution. Note that PCOM1 accounts for ~96.6% of variation over the entire spectral range and follows faithfully the NIR-OAS of glucose, whereas PCOM2 exhibits random behavior. **(B)** Estimated *C_gl_* values for a probe set using ratiometric (red dots) and sparse dictionary learning (SDL) method (blue triangles) with a solid line representing expected *C_gl_* from PS values. Note tighter correlation of expected *C_gl_* with *C_gl_* calculated by SDL.

To represent more quantitatively the OAS of glucose, the first two components identified in the PCA analysis of the raw optoacoustic spectra were plotted graphically (Figure 4A). For illustration purposes and ready cross-comparison, the Δ*I*_OA_ of 169 mg/dl glucose solution is also plotted on Figure 4A. The variance of the first PCA component (PCOM1) accounts for ~96.6% of total variation, while PCOM2 accounted for a negligible ~1.6% variation. PCOM3 (not shown) offered ~0.8% variation and subsequent components were negligible. PCOM1 is found to faithfully follow the Δ*I*_OA_ trajectory shown in Figure 3A, while the data points of PCOM2 appear to have random values and possibly reflect systematic errors in the measurement (Figure 4A). This observation and the calculated SDs of components indicate that only PCOM1 was significant for glucose measurements. Unidimensional composition of the titration dataset indicates that under our settings, within investigated concentration ranges, changes of the intensity of OAS obeyed the Beer–Lambert law, and thus the dataset over the entire concentration range can be reconstructed based on PCOM1 only.

To determine if the SDL or ratiometric methods present more authentically changes in glucose concentration revealed with PCA method, we compared the glucose concentration estimated by the SDL and ratiometric methods applied on PCA-filtered data against the expected titration values. Ratiometric measurements were performed at 1,250 and 1,650 nm wavelengths that exhibited the highest spectral intensity change (Δ*I*_OA_). Parameters of *R_gl_* (0.74) and *K* (403.3) were calculated in relation to Eq. 2 using training set calibration. Figure 4B summarizes the mean and SD of *C_gl_* data defined with ratiometry and SDL overlaid with the expected glucose concentrations from the probe set. While both methods reproduced an increase in glucose concentration as a function of titration, the average SD and SNR for all data points assessed with SDL method was notably higher (SNR = 40.2, SD = 8.6; *n* = 30) and revealed better correlation (*r* = 0.96) compared to those determined with ratiometric method (SNR = 10.3, SD = ± 15.8, and *r* = 0.91; *n* = 35) (Figure 4B).

## Discussion

In this study, we introduced an OA spectroscope and characterized the optoacoustic spectra of glucose in the extended NIR region (900–1,900 nm). While the detection of intensity variations in an interrogating OS beam can be very sensitive, ultrasound detectors and the generation of ultrasound waves in tissues may be regarded as less efficient to the optical counterpart. The key advantage of acquiring ultrasound waves (versus optical signals), however, is less sensitivity of sound waves to scattering with better penetration, which holds major promises for non-invasive imaging and glucose sensing in deep tissue. We investigated if optoacoustic measurements attain sufficient sensitivity in detecting glucose and demonstrated herein for the first time optoacoustic detection of glucose NIR spectra at physiologically relevant concentrations in aqueous media. A linear dependence between the OA signal intensity and glucose concentrations (0–170 mg/dl) was explicitly shown, confirming that optoacoustic measurements attain sufficient sensitivity in the extended NIR spectral region, i.e., the 850–1,900 nm range. Our measurements and analytical methods are indifferent to the intra-assay variations and provide a comprehensive characterization of the extended NIR optoacoustic glucose spectrum, which could serve for reference purposes.

We applied and compared two alternative methods for extracted glucose spectra. The first approach relied on dictionary learning method which defines the basis dictionary—essentially the Δ*I*_OA_ spectra of unit concentration of analyte finding least-square solution of set of liner equations. This dictionary is then used to estimate *C_gl_*-s of probe the set. Although it yielded SNR ~3.9-fold higher than the second (ratiometric) method with better correlation to pre-defined unit spectra (*r* = 0.96), the accuracy of this method depends on precise definition of the baseline as well as the eigenspectra computed from the titration set, and requires careful calibration for measurements in more complex environment. The second, ratiometric approach, was based on optoacoustic readouts at 1,250 and 1,650 nm. While both the SNR and correlation coefficient of ratiometric measurements were somewhat lower than in case of SDL method, the main benefit of this approach is its reliance on the data generated at two wavelengths, which speeds up the measurements and lowers their costs. Last but not least, because the absolute values of *I*_OA_ are used directly for ratiometric analysis, all calculations related to baseline subtraction and possible estimation errors are minimized.

The optoacoustic spectrum of glucose determined herein is in general agreement with the OS measurements reported elsewhere with optimal wavelengths for glucose measurements matching to reported 1,000–1,300 nm and 1,500–1,900 nm (20–22). The differential glucose spectra [Δ*I*_OA_(λ)] peaks at 1,650 nm, which is in line with glucose absorption spectra obtained by FT NIR from higher *C_gl_* (20). The positive maximum of OAS of glucose fits between two (1,536 and 1,688 nm) NIR overtones of glucose (21) while the negative peak of Δ*I*_OA_ matches with three dominant overtones of water (880, 980, and 1,211 nm) and come at 900–1,300 nm range, also in general agreement with published data. Finally, the Δ*I*_OA_ of our OAS measurements shows a valley around 1,450 nm, which is also consistent with previously reported negative peak of glucose defined with OS (20). Within this region, two overtones corresponding to water (1,454 nm) and glucose (1,408 nm) absorption appear to negate each other (23). It should be noted that these parameters are specific to glucose spectra measurements in aqueous medium. Interestingly, the absorption of glucose and water within these wavelengths are comparable, as evident from autocorrelation of FT-IR spectroscopic maps, which showed that changes driving the resultant spectra in opposite directions are in-phase variation with each other (22). As a result, the region at 1,400–1,450 nm is insensitive to glucose titration and can be used as a reference for estimating the background absorption of turbid medium. Finally, the small peak at 1,350–1,375 nm on both titration spectra (Figure 3A) and PCOM1 score (Figure 4A) matches to the region of the predominance of glucose absorption (21). It is essential to note that the SNR levels for all acquired OAS were above 11.4 and can be accounted to great extent by the energy fluctuations of tunable laser. As such, the SNR could be further increased by compensation for laser-related variability and use of more stable optical source.

Among non-invasive techniques proposed for *in vivo* glucose measurements, those using spectroscopic modalities, such as fluorescence spectroscopy, NIR, or MIR spectroscopies show clear promise (20, 22, 24, 25). However, the pending weaknesses of the pure optical methods related to photon scattering impede accurate spectral discrimination and quantification (26, 27). Thus, there is pressing need in rigorous quantitative methods for assessment of glucose levels within the physiological ranges, for both basic and translational applications. As the ubiquitous fuel in biology and the prime energy source in most organisms (including humans), glucose is used in either anaerobic or aerobic respirations, with disruption of glucose metabolism implicated in a range of metabolic disease, including diabetes. Described herein OA measurements of physiological concentrations of glucose in aqueous solution in the extended NIR range conceals potentials of overcoming the limitations of optical spectroscopic methods. Introduced in this study, OA spectroscope along with presented proof of concept experimental measurements to provide a framework for the development of rigorous OAS tools capable of glucose detection and its accurate quantification *ex vivo* and, potential translation *in vivo*. Such advance should facilitate non-invasive measurements of glucose under both laboratory and clinical settings, with a prospect of improving the management and prognosis of diabetes and related disorders.

## Author Contributions

AG and VN designed the study. AG conducted experimental measurements and data analysis. AG and SO drafted the manuscript and prepared the figures. All authors read and approved the final version of the manuscript. Authors have no conflict of interest to report.

## Conflict of Interest Statement

The authors declare that the research was conducted in the absence of any commercial or financial relationships that could be construed as a potential conflict of interest.
